# Supplementary material for: Sex Difference in Effectiveness of Early Rhythm- over Rate-Control in Patients with Atrial Fibrillation
Source: J Clin Med. 2022 Aug 25;11(17):4991. doi: 10.3390/jcm11174991 (PMC9456685; doi:10.3390/jcm11174991)

Supplementary Materials

Supplementary Table S1. Summary of strategies for emulating target trial

Components	**Target trial (EAST-AFNET4)**	**This study**
**Inclusion period**	28 July 2011 – 30 December 2016	1 January 2005 – 31 December 2015
**Eligibility criteria**	1) Adults (≥18 years of age) who were older than 75 years of age, had had a previous transient ischemic attack or stroke, or met two of the following criteria: age greater than 65 years, female sex, heart failure, hypertension, diabetes mellitus, severe coronary artery disease, chronic kidney disease, and left ventricular hypertrophy<br>2) Early AF (diagnosed ≤12 months before enrolment)	1) Selected adults (≥18 years of age) that received a rhythm-control or rate-control treatments within 12 months after AF diagnosis<br>2) Participants have no prior history of prescriptions and no records of ablation in the database who were older than 75 years of age, had a previous transient ischemic attack or stroke, or met two of the following criteria: age greater than 65 years, female sex, heart failure, hypertension, diabetes mellitus, myocardial infarction, and chronic kidney disease<br>3) Undergoing oral anticoagulation (>90 days of supply within 180 days after their first recorded prescription of rhythm- or rate-control medications or ablation procedure)
**Exposed group**	Rhythm control: AADs, AF ablation, cardioversion of persistent AF, to be initiated early after randomization	Rhythm control: a prescription of more than a 90-day supply of any rhythm–control drugs in the 180-day period since the first prescription or the performance of an ablation procedure for AF.
**Unexposed group**	Usual care: initially treated with rate-control therapy without rhythm-control therapy	Rate control: a prescription of more than a 90-day supply of any rate-control drugs in the 180-day period since the first prescription and with no prescription of rhythm-control drug and no ablation within this period. Patients prescribed rhythm-control drugs for more than 90 days or who underwent ablation within the 180-day period since the initiation of rate-control drugs were classified as intention–to–treat with rhythm control.
**Primary outcome**	1) A composite of death from cardiovascular causes, stroke, or hospitalization with worsening of heart failure or acute coronary syndrome<br>2) The number of nights spent in the hospital per year.	A composite of death from cardiovascular causes, ischemic stroke, hospitalization for heart failure, or acute myocardial infarction
**Secondary outcome**	Each component of the primary outcome, rhythm, left ventricular function, quality of life, AF-related symptom	Each component of the primary outcome

**Safety outcome**	A composite of death from any cause, stroke, or pre-specified serious adverse events of special interest capturing complications of rhythm-control therapy	A composite of death from any cause, intracranial or gastrointestinal bleeding requiring hospitalization, or pre–specified serious adverse events of special interest capturing complications of rhythm-control therapy
**Follow-up**	From randomization until the end of the trial, death, or withdrawal from the trial.	From 180 days after their first recorded prescription or procedure to avoid immortal time bias until the end of follow-up of the database (31 December 2016) or death.

AAD, antiarrhythmic drug; AF, atrial fibrillation.

Supplementary Table S2. Definitions and ICD–10 codes used for defining comorbidities, rate- and rhythm-control methods for atrial fibrillation

Comorbidities	Definitions	ICD-10 codes or conditions
Atrial fibrillation	Defined from diagnosis[a]	I48
Heart failure	Defined from diagnosis[a]	ICD-10: I11.0, I50, I97.1
Previous hospitalization for heart failure	Defined from principal or first secondary admission diagnoses of heart failure	ICD-10: I11.0, I50, I97.1
Hypertension	Defined from diagnosis[a] plus treatment	I10, I11, I12, I13, I15<br>Treatment: all kinds of blood pressure lowering medications (>1 month).
Diabetes mellitus	Defined from diagnosis[a] plus treatment	E10, E11, E12, E13, E14<br>Treatment: all kinds of oral antidiabetics and insulin
Dyslipidemia	Defined from diagnosis[a]	E78
Ischemic stroke	Defined from diagnosis[a]	ICD-10: I63, I64
Transient ischemic attack	Defined from diagnosis[a]	ICD-10: G45
Hemorrhagic stroke	Defined from diagnosis[a]	ICD-10: I60, I61, I62
Myocardial infarction	Defined from diagnosis[a]	ICD-10: I21, I22, I25.2
Peripheral arterial disease	Defined from diagnosis[a]	I70, I71
Valvular heart disease	Defined from diagnosis[a] mitral stenosis or claims for heart valve surgery	ICD-10: I05.0, I05.2, I34.2, Z95.2–4<br>Claim for valve replacement or valvuloplasty: O1781, O1782, O1783, O1791, O1792, O1793, O1797, O1794, O1795, O1796, O1798
Chronic kidney disease	Defined from eGFR (if laboratory value was not available, diagnosis code was used)	eGFR <60 mL/min per 1.73 m^2<br>N18, N19
**Rate- and rhythm control for atrial fibrillation (available in South Korea)**	**Definitions**	**ICD-10 codes or conditions**
**Rate control**		
Beta-blocker	atenolol, bisoprolol, carvedilol, metoprol, nebivolol, propranolol, labetalol	
Calcium channel blocker	diltiazem, verapamil	
Cardiac glycosides	digoxin	
**Rhythm control**		
Class Ic	flecainide, pilsicainide, propafenone	
Class III	amiodarone,	

	dronedarone, sotalol	
Catheter ablation for AF	Defined from admission diagnosis of AF plus claims for ablation procedures	ICD-10: I48<br>Claim codes: M6542 (Conventional Radiofrequency Ablation of Atrial fibrillation) or M6547 (Radiofrequency Ablation of Atrial fibrillation Through Intracardiac Electrophysiologic 3-Dimensional Mapping)
Cardioversion	Defined from diagnosis of AF plus claims for cardioversion	ICD-10: I48<br>Claim codes:M5880

[a] To ensure accuracy, diagnosis was established based on one inpatient or two outpatient records of ICD-10 codes in the database.

AF, atrial fibrillation; ICD, International Classification of Diseases-10th revision; eGFR, estimated glomerular filtration rate

Supplementary Table S3. Definitions and ICD-10 codes used for defining study outcomes

Outcomes	Definitions	Codes or conditions	PPV
**Primary composite outcome**			
Cardiovascular death[a]			
Ischemic stroke	Defined from admission diagnosis with concomitant imaging studies of the brain or related death	ICD-10: I63, I64	90.6%[b] (2347/2591)
Hospitalization for heart failure	Defined from principal or first secondary admission diagnoses of heart failure	ICD-10: I11.0, I50, I97.1	82.1%[b] (110/134)
Acute myocardial infarction	Defined from admission diagnosis of acute myocardial infarction concurrently with coronary angiography or related death	ICD-10: I21, I22	86.5%[c] (4054/4688)
**Safety outcomes**			
All–cause death[d]			
Intracranial bleeding	Defined from admission diagnosis with concomitant imaging studies of the brain or related death	ICD-10: I60–I62	87.5%[b] (286/327)
Gastrointestinal bleeding	Defined from admission diagnosis or related death	ICD-10: K25–28 (subcodes 0–2 and 4–6 only), K62.5, K92.0, K92.1, K92.2, I85.0, I98.3	92.0%[e] (184/200)
Serious adverse events related to rhythm control			
Cardiac tamponade	Defined from claims for pericardiocentesis	Claim codes:C8060, C8061	–
Syncope	Defined from either one diagnosis during hospitalization or more than twice at outpatient clinics	ICD-10: R55.x	–
Sick sinus syndrome	Defined from either one diagnosis during hospitalization or more than twice at outpatient clinics	ICD-10: I495.	91.1%[b] (307/337)
Atrioventricular block	Defined from either one diagnosis during hospitalization or more than twice at outpatient clinics	ICD-10: I44.1, I44.2, I44.3, I45.3, I45.8, I45.9	95.7%[b] (264/276)
Pacemaker implantation	Defined from claims for pacemaker implantation	Claim codes: O2003, O2004, O0203, O0204, O0205, O0206, O0207	-
Sudden cardiac arrest	Defined from admission diagnosis or related death[f]	ICD-10: I46, I49.0	80.2%[g] (586/731)

PPV was represented as % (number of true positive cases / number of examined cases)
[a]Defined as a death mainly due to ischemic stroke, heart failure, or acute myocardial infarction, similar to our previous studies (Kim, D. et al. Risk of dementia in stroke-free patients diagnosed with atrial fibrillation: data from a population–based cohort. Eur Heart J. 2019;40(28):2313–2323.; Yang, PS. et al. Changes in Cardiovascular Risk Factors and Cardiovascular Events in the Elderly Population. J Am Heart Assoc. 2021;10(11):e019482.)

[b]Authors conducted a validation study using hospital administrative data from two tertiary hospitals

[c]Validated in a study by Lee, HY. et al. (Atrial fibrillation and the risk of myocardial infarction: a nation-wide propensity-matched study. Sci Rep 2017;7(1):12716)

[d]Information on death (date and causes) was confirmed from the National Population Registry of the Korea National Statistical Office, which conducts central registration of death based on death certificates. It is a national agency covering the entire Korean population

[e]Validated in a study by Park, J. et al. (Validation of diagnostic codes of major clinical outcomes in a National Health Insurance database. Int J Arrhythm 2019;20:5)

[f]To avoid erroneous inclusion of the patients with non–cardiac arrest, we excluded the patient with sudden arrest diagnosis accompanied by respiratory arrest (R09.0, R09.2), gastrointestinal bleeding (I85.0, K25.0, K25.4, K26.0, K26.4, K27.0, K27.4, K92.0–K92.2), brain hemorrhage (I60.x–I62.x, S06.4–S06.6), septic shock (A41.9, R57.2), pregnancy and delivery (O00–O99), diabetic ketoacidosis (E14.1), anaphylaxis (T78.2), and accidents including suicide (T71, T75.1, T36–T65, V80–V89, W76.x, X60–X84)

[g]Validated in a study by Kim, IJ. et al. (Relationship Between Anemia and the Risk of Sudden Cardiac Arrest – A Nationwide Cohort Study in South Korea. Circ J 2018;82(12):2962–9)

Supplementary Table S4. Relative effect of rhythm over rate control on safety outcomes after overlap weighting

	Men				Women				
	IR	IR	Hazard ratio (95% CI)	*P*-value	IR	IR	Hazard ratio (95% CI)	*P*-value	*P* for interaction
**AF treatment (<6 months since the first diagnosis of AF)**									
	Rhythm control **(N=2123)**	Rate control **(N=2123)**			Rhythm control **(N=1912)**	Rate control **(N=1912)**			
Composite safety outcome	7.97	8.20	0.97 (0.90–1.05)	0.471	8.36	7.62	1.10 (1.01–1.19)	0.027	0.040
All-cause death	4.60	5.27	0.87 (0.79-0.96)	0.005	4.38	4.56	0.96 (0.87–1.06)	0.443	0.175
Intracranial bleeding	0.59	0.78	0.78 (0.60-1.01)	0.057	0.74	0.75	0.99 (0.77–1.27)	0.954	0.185
Gastrointestinal bleeding	1.67	2.01	0.85 (0.72-0.99)	0.042	1.74	1.97	0.89 (0.76–1.05)	0.156	0.671
Serious adverse event related to rhythm control	2.91	2.15	1.37 (1.20–1.57)	<0.001	3.36	2.00	1.68 (1.46–1.94)	<0.001	0.041
Cardiac tamponade	0.09	0.06	1.68 (0.76–3.72)	0.199	0.07	0.03	2.64 (1.02–6.83)	0.046	0.511
Syncope	1.23	1.04	1.20 (0.98–1.47)	0.071	1.30	0.97	1.34 (1.09–1.65)	0.005	0.445
Sick sinus syndrome	0.67	0.15	4.44 (2.97–6.64)	<0.001	1.05	0.37	2.86 (2.12–3.86)	<0.001	0.083
Atrioventricular block	0.47	0.19	2.51 (1.70–3.72)	<0.001	0.43	0.24	1.83 (1.25–2.67)	0.002	0.252
Pacemaker implantation	0.39	0.11	3.75 (2.26–6.22)	<0.001	0.49	0.23	2.12 (1.44–3.11)	<0.001	0.078
Sudden cardiac arrest	0.50	0.63	0.81 (0.61–1.06)	0.126	0.56	0.43	1.29 (0.95–1.75)	0.099	0.024
**AF treatment (6–12 months since the first diagnosis of AF)**									
	Rhythm control **(N=132)**	Rate control **(N=132)**			Rhythm control **(N=100)**	Rate control **(N=100)**			
Composite safety outcome	7.82	9.26	0.85 (0.63–1.14)	0.279	7.67	6.04	1.27 (0.89–1.82)	0.196	0.093
All-cause death	4.50	6.66	0.67 (0.47–0.95)	0.025	2.82	3.43	0.82 (0.50–1.34)	0.427	0.525
Intracranial bleeding	0.81	0.90	0.96 (0.40–2.28)	0.925	1.13	0.81	1.41 (0.61–3.26)	0.424	0.541
Gastrointestinal bleeding	1.26	3.05	0.44 (0.24–0.78)	0.005	1.42	1.19	1.18 (0.56–2.47)	0.658	0.034
Serious adverse event related to rhythm control	3.60	2.51	1.55 (0.94–2.55)	0.087	2.70	1.80	1.52 (0.79–2.91)	0.211	0.959
Cardiac tamponade	0.06	0.12	0.50 (0.04–5.66)	0.572	0.05	0	-	-	-
Syncope	1.69	1.13	1.60 (0.77–3.33)	0.207	1.57	0.86	1.87 (0.76–4.63)	0.176	0.799
Sick sinus syndrome	0.40	0.21	2.05 (0.57–7.40)	0.274	1.23	0.54	2.35 (0.76–7.29)	0.139	0.888
Atrioventricular block	0.49	0.15	3.42 (0.70–16.78)	0.129	0.33	0.49	0.69 (0.13–3.57)	0.661	0.174
Pacemaker implantation	0.39	0.03	16.81 (1.95–144.90)	0.010	0.32	0.28	1.14 (0.25–5.23)	0.862	0.047
Sudden cardiac arrest	0.83	0.88	1.00 (0.42–2.41)	0.997	0.08	0.12	0.66 (0.10–4.27)	0.661	0.692

AF, atrial fibrillation; CI, confidence interval; IR, incidence rate.

Supplementary Table S5. The relative effect of rhythm control over rate control on primary composite outcome in men and women after 1:1 propensity score matching

Primary composite outcome	Number of events	Person-years	IR	Number of events	Person-years	IR	Absolute rate difference per 100 person-years (95% CI)	Hazard ratio (95% CI)	*P*-value	*P* for interaction
**AF treatment (<6 months since the first diagnosis of AF)**										0.935
**Men**	Rhythm control **(N=3097)**			Rate control **(N=3097)**						
	676	11558	5.85	752	11093	6.78	-0.93 (-1.59 to -0.27)	0.89 (0.80–0.98)	0.014	
**Women**	Rhythm control **(N=2711)**			Rate control **(N=2711)**						
	735	10175	7.22	813	10013	8.12	-0.90 (-1.66 to -0.13)	0.89 (0.81–0.98)	0.025	
**AF treatment (6–12 months since the first diagnosis of AF)**										0.007
**Men**	Rhythm control **(N=158)**			Rate control **(N=158)**						
	30	615	4.88	48	556	8.63	-3.75 (-6.76 to -0.75)	0.62 (0.39–0.98)	0.040	
**Women**	Rhythm control **(N=109)**			Rate control **(N=109)**						
	35	409	8.56	25	459	5.45	3.11 (-0.44 to 6.66)	1.59 (0.95–2.66)	0.077	

AF, atrial fibrillation; CI, confidence interval; HR, hazard ratio; IR, incidence rate.

Supplementary Table S6. The relative effect of anti–arrhythmic drugs over rate control on primary composite outcome in men and women according to timing of treatment initiation after overlap weighting

**Primary composite outcome**	Number of events	Person–years	IR	Number of events	Person–years	IR	Absolute rate difference per 100 person–years (95% CI)	Hazard rati* (95% CI)	*P*-value	*P* for interaction
**AF treatment (<6 months since the first diagnosis of AF)**										0.819
**Men**	Rhythm control **(N=2119)**			Rate control **(N=2119)**						
	461	7875	5.86	520	7567	6.87	-1.01 (-1.81 to -0.22)	0.87 (0.79–0.94)	0.001	
**Women**	Rhythm control **(N=1910)**			Rate control **(N=1910)**						
	516	7187	7.18	590	6946	8.49	-1.30 (-2.23 to -0.38)	0.85 (0.79–0.93)	<0.001	
**AF treatment (6~12 months since the first diagnosis of AF)**										0.023
**Men**	Rhythm control **(N=130)**			Rate control **(N=130)**						
	30	513	5.91	39	460	8.42	-2.50 (-5.89 to 0.88)	0.74 (0.54–1.03)	0.074	
**Women**	Rhythm control **(N=99)**			Rate control **(N=99)**						
	33	390	8.44	26	402	6.50	1.94 (-1.87 to 5.75)	1.32 (0.92–1.88)	0.137	

AF, atrial fibrillation; CI, confidence interval; HR, hazard ratio; IR, incidence rate.

Supplementary Table S7. Risk of 24 falsification endpoints in weighted male and female patients undergoing rhythm control compared with rate control

Endpoints	<6months				6–12 months			
	Men		Women		Men		Women	
	Hazard ratio (95% CI)	*P*-value	Hazard ratio (95% CI)	*P*-value	Hazard ratio (95% CI)	*P*-value	Hazard ratio (95% CI)	*P*-value
Influenza	0.76 (0.57–1.01)	0.054	0.83 (0.64–1.09)	0.180	0.57 (0.15–2.07)	0.389	1.64 (0.43–6.28)	0.470
Major fracture	0.98 (0.79–1.21)	0.838	1.08 (0.94–1.24)	0.257	0.76 (0.34–1.70)	0.510	0.78 (0.41–1.45)	0.428
Urinary tract infection	1.04 (0.94–1.14)	0.451	0.98 (0.92–1.06)	0.679	1.33 (0.97–1.84)	0.079	1.04 (0.77–1.41)	0.810
Syphilis	0.82 (0.49–1.35)	0.429	0.79 (0.48–1.29)	0.348	3.94 (0.46–34.01)	0.212	7.06 (0.81–61.74)	0.077
Viral enteritis	1.20 (0.80–1.82)	0.380	0.88 (0.61–1.27)	0.496	0.94 (0.27–3.25)	0.923	1.91 (0.47–7.73)	0.366
Warts	1.46 (0.85–2.50)	0.172	0.93 (0.52–1.66)	0.809	0.54 (0.09–3.21)	0.496	0.41 (0.04–4.85)	0.483
Acute hepatitis A	1.17 (0.63–2.16)	0.620	1.09 (0.59–2.01)	0.785	2.18 (0.27–17.44)	0.462	0.55 (0.10–3.02)	0.488
Stomach cancer	0.81 (0.64–1.04)	0.092	0.89 (0.63–1.27)	0.524	0.58 (0.25–1.34)	0.201	1.01 (0.22–4.58)	0.988
Lipoma	1.14 (0.80–1.64)	0.473	1.34 (0.86–2.09)	0.195	0.80 (0.22–2.89)	0.729	0.69 (0.12–3.84)	0.669
Carpal tunnel syndrome	1.30 (0.84–2.00)	0.240	0.93 (0.68–1.29)	0.674	0.74 (0.14–3.89)	0.720	0.52 (0.13–2.08)	0.358
Pterygium	1.08 (0.78–1.48)	0.651	1.15 (0.86–1.54)	0.354	0.90 (0.24–3.40)	0.877	1.05 (0.31–3.60)	0.940
Meniere's disease	1.15 (0.87–1.51)	0.328	1.06 (0.84–1.34)	0.633	1.51 (0.56–4.10)	0.416	2.87 (0.92–8.97)	0.069
Benign paroxysmal positional vertigo	1.16 (0.93–1.45)	0.197	1.11 (0.94–1.33)	0.224	1.13 (0.54–2.38)	0.748	0.80 (0.38–1.71)	0.570
Varicose veins of lower extremities	0.99 (0.70–1.38)	0.937	0.74 (0.50–1.11)	0.147	0.50 (0.14–1.78)	0.287	1.08 (0.26–4.46)	0.920
Acute appendicitis	1.41 (0.88–2.27)	0.154	1.15 (0.75–1.76)	0.536	0.42 (0.09–1.96)	0.272	0.80 (0.17–3.67)	0.770
Diverticulitis of intestine	1.35 (0.89–2.06)	0.160	1.04 (0.66–1.62)	0.873	0.52 (0.16–1.70)	0.279	0.51 (0.09–2.99)	0.456
Cellulitis	1.11 (0.99–1.23)	0.060	1.05 (0.93–1.17)	0.435	0.98 (0.65–1.48)	0.916	0.63 (0.40–1.01)	0.053
Urticaria	1.05 (0.96–1.15)	0.330	1.05 (0.96–1.15)	0.292	1.02 (0.72–1.44)	0.911	0.80 (0.56–1.16)	0.245
Ingrowing nail	1.33 (0.96–1.83)	0.083	1.12 (0.84–1.50)	0.423	0.43 (0.09–1.91)	0.265	1.18 (0.20–7.10)	0.855
Frozen shoulder	1.06 (0.94–1.20)	0.346	0.94 (0.84–1.05)	0.278	1.21 (0.73–1.99)	0.467	0.97 (0.61–1.56)	0.907
Osteomyelitis	0.79 (0.46–1.38)	0.415	0.76 (0.36–1.60)	0.468	3.16 (0.29–34.3)	0.345	6.78 (0.44–105.4)	0.171
Dysuria	1.02 (0.87–1.19)	0.828	0.94 (0.80–1.11)	0.448	0.92 (0.53–1.58)	0.753	0.67 (0.34–1.29)	0.230
Burns	1.15 (0.90–1.48)	0.270	0.78 (0.79–1.21)	0.839	2.05 (0.83–5.05)	0.121	0.86 (0.40–1.88)	0.712
Anaphylaxis/Allergic reaction	1.02 (0.73–1.43)	0.900	0.77 (0.54–1.12)	0.170	1.29 (0.33–5.12)	0.713	1.08 (0.26–4.50)	0.916

CI, confidence interval

Supplementary Figure S1. Distributions of the propensity scores in men (A) and women (B) before and after overlap weighting.

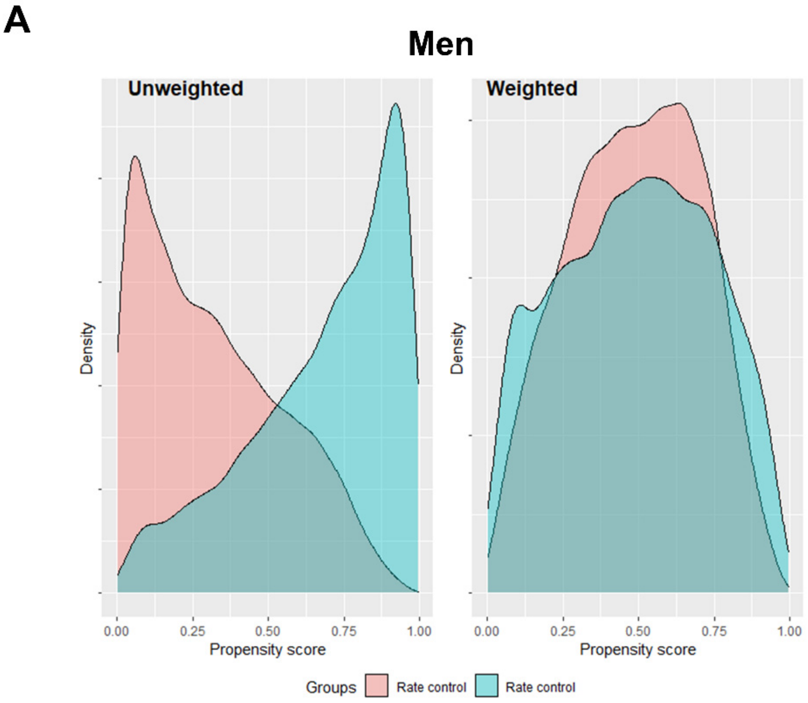

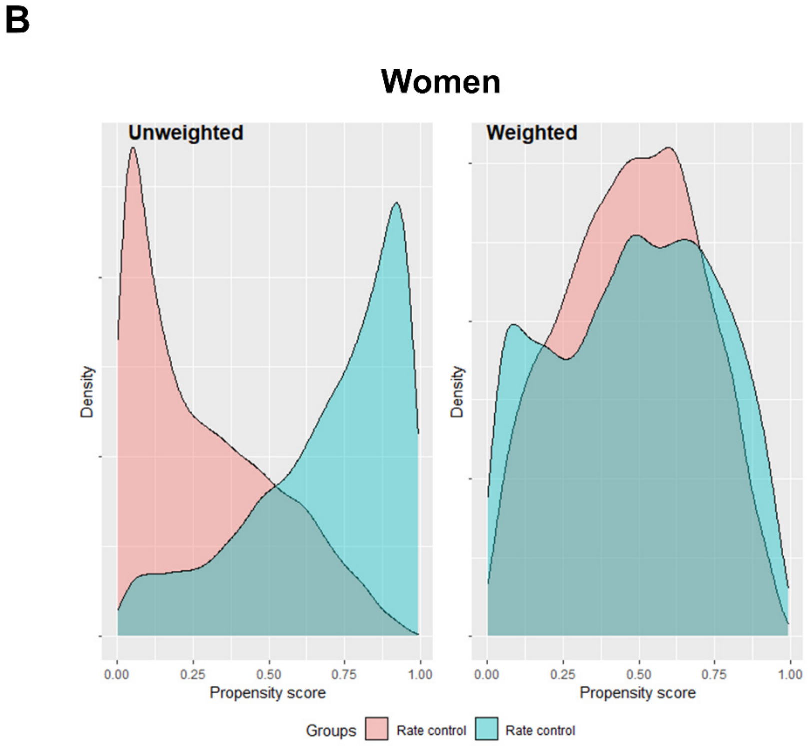

Supplement: Supplementary file 1 [file jcm-11-04991-s001.zip › jcm-1839153-supplementary.pdf]
